# Genetic Mapping and Comparative Expression Analysis of Transcription Factors in Cotton

**DOI:** 10.1371/journal.pone.0126150

**Published:** 2015-05-06

**Authors:** Xuemei Chen, Xin Jin, Ximei Li, Zhongxu Lin

**Affiliations:** National Key Laboratory of Crop Genetic Improvement, Huazhong Agricultural University, Wuhan 430070, Hubei, China; USDA-ARS-SRRC, UNITED STATES

## Abstract

Transcription factors (TFs) play an important role in the regulation of plant growth and development. The study of the structure and function of TFs represents a research frontier in plant molecular biology. The findings of these studies will provide significant information regarding genetic improvement traits in crops. Currently, a large number of TFs have been cloned, and their function has been verified. However, relatively few studies that genetically map TFs in cotton are available. To genetically map TFs in cotton in this study, specific primers were designed for TF genes that were published in the Plant Transcription Factor Database. A total of 977 TF primers were obtained, and 31 TF polymorphic loci were mapped on 15 cotton chromosomes. These polymorphic loci were clearly preferentially distributed on chromosomes 5, 11, 19 and 20; and TFs from the same family mapped to homologous cotton chromosomes. *In-silico* mapping verified that many mapped TFs were mapped on their corresponding chromosomes or their homologous chromosomes’ corresponding chromosomes in the diploid genomes. QTL mapping for fiber quality revealed that TF-Ghi005602-2 mapped on Chr19 was associated with fiber length. Eighty-five TF genes were selected for RT-PCR analysis, and 4 TFs were selected for qRT-PCR analysis, revealing unique expression patterns across different stages of fiber development between the mapping parents. Our data offer an overview of the chromosomal distribution of TFs in cotton, and the comparative expression analysis between *Gossypium hirsutum* and *G*. *barbadense* provides a rough understanding of the regulation of TFs during cotton fiber development.

## Introduction

Transcription factors (TFs) are a class of the most widely studied and important trans-acting factors, regulating gene expression at the transcriptional level. A typical TF from a higher-order plant typically contains a DNA-binding domain, a transcription regulation domain, an oligomerization site, and a nuclear localization domain [1]. TFs play an important role in the regulation of plant growth and development, organ morphogenesis, secondary metabolism, hormonal signal transduction, and plant responses to various environmental stresses [2–7]. Therefore, it is likely that investigating and exploiting the function of TFs could provide an alternative approach for improving cotton fiber quality and production.

To date, a large number of TFs have been reported, and their functions have been successively investigated and verified in cotton. Among them, the majority of research on TFs has focused on the ethylene-response factor (ERF) family, myeloblastosis (MYB) family, WKRY family, and basic helix-loop-helix (bHLH) family. A large amount of research has confirmed that the MYB family plays a functional role in cotton fiber differentiation and development [8–13]. ERF genes are induced by biotic and abiotic stresses in cotton and are involved in regulating plant disease resistance pathways [14,15]. The bHLH family plays an important role in regulating plant secondary metabolism [16,17] and morphogenesis [6,7,18]. Plant WKRY TFs have important functions in the transcriptional regulation of a variety of biological processes that are related to growth and development [19–21], various environmental stimuli [22–24], and disease resistance pathways [25–27].

As of April 2012, approximately 1,116 TFs from 50 families were annotated in *Gossypium hirsutum* (http://planttfdb.cbi.edu.cn/index.php?sp=Ghi). However, previous research has focused mainly on identifying and verifying the biological functions of these TFs rather than their genomic distributions. In this study, the TFs were mapped to reveal their genomic distribution in cotton using specific primers designed based on transcription factor sequences available in the Plant Transcription Factor Database (http://planttfdb.cbi.edu.cn/). We also confirmed their chromosomal location by in-silico mapping the experimental mapped TFs in two sequenced diploid genomes, A_2_ genome of *G*. *arboretum* and D_5_ genome of *G*. *raimondii*. QTL mapping was conducted to identify TFs related to fiber quality. RT-PCR and qRT-PCR analysis were also conducted to detect differences in expression during fiber development between *G*. *hirsutum* and *G*. *barbadense* in selected TFs from each family.

## Materials and Methods

### Plant materials

Polymorphisms of the designed TF primers were detected using *G*. *hirsutum* cv. Emian22 and *G*. *barbadense* acc. 3–79, which are the parents of the BC_1_ mapping population [(Emian22 × 3–79) × Emian22] [28,29], using single-strand conformation polymorphism (SSCP) with minor modifications [30]. The BC_1_ population, which consisted of 141 plants, was used as the mapping population for all of the polymorphic TF markers.

### Marker development

**Fig 1:**
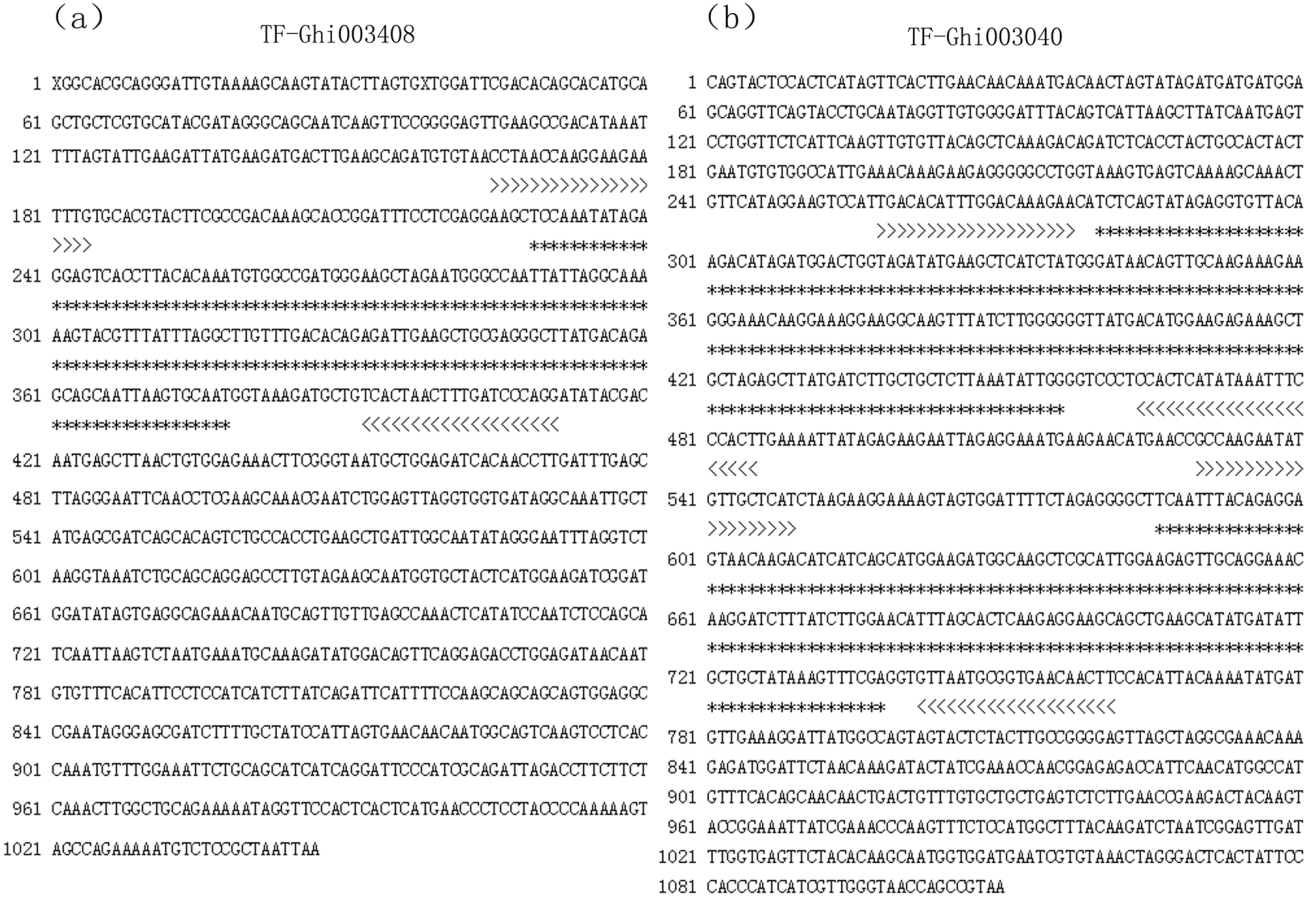
Primer design strategies for TFs. (a) TFs with one motif (GenBank acc No. Ghi003408); (b) TFs with two motifs (GenBank acc No. Ghi003040). *: Target sequence; >>>>>: Primer region.

Cotton TFs were obtained from the Plant Transcription Factor Database V2.0 (http://planttfdb.cbi.edu.cn/), which contains 1,116 *G*. *hirsutum* TFs classified into 50 families (Table 1). The primers were designed based on the sequences surrounding specific motifs of the TF genes using Primer 3.0 (http://frodo.wi.mit.edu/primer3/). For those TFs with multiple motifs, if the sequence interval between motifs was too long, individual primers were designed for each motif (examples are presented in Fig 1). The criteria for the primer design were as follows: a primer length of 18 to 25 bp (20 bp is optimal), a GC content of 35 to 70% (50% is optimal), an annealing temperature of 50 to 65°C (55°C is optimal), and a PCR product size ranging from 100 to 1,000 bp. The primers were named “TF-Ghi ××××××”. For TFs with multiple motifs, the primers were named “TF-Ghi ××××××-1” and “TF-Ghi ××××××-2”.

**Table 1:** Distribution of TFs in various families.

Family	No.	Family	No.	Family	No.	Family	No.	Family	No.
AP2	8	ARF	7	ARR-B	2	B3	23	BBR/BPC	6
BES1	7	C2H2	56	C3H	44	CAMTA	2	CO-like	4
CPP	2	DBB	12	Dof	22	E2F/DP	3	EIL	10
ERF	112	FAR1	7	G2-like	31	GATA	24	GRAS	46
GRF	13	GeBP	5	HB-other	15	HD-ZIP	40	HSF	17
LBD	14	M-type	2	MIKC	21	MYB	75	MYB_related	59
NAC	50	NF-YA	5	NF-YB	10	NF-YC	11	Nin-like	7
RAV	1	S1Fa-like	2	SBP	13	SRS	4	TALE	14
TCP	23	Trihelix	38	VOZ	5	WOX	6	WRKY	55
Whirly	1	YABBY	4	ZF-HD	15	bHLH	92	bZIP	71

### PCR amplification and electrophoresis

Polymerase chain reaction (PCR) of the TF markers was performed in 10 μL of solution containing 25 ng DNA template, 1 × buffer, 2.0 mmol L^-1^ MgCl_2_, 0.25 mmol L^-1^ dNTPs, 0.16 μmol L^-1^ forward primer, 0.16 μmol L^-1^ reverse primer, 0.8 units Taq DNA polymerase, and ddH_2_O to a final volume of 10 μl. PCR was performed using the following parameters: 95°C for 5 min; 34 cycles of 94°C for 50 sec, 56°C for 45 sec, and 72°C for 60 sec; and a final extension of 5 min at 72°C. The PCR products were then separated in an 8% non-denaturing polyacrylamide gel at a constant voltage of 15 W for approximately 4 h at room temperature. After electrophoresis, all of the DNA fragments were visualized by silver staining.

### Experimental mapping and identification of TFs related to fiber quality

The polymorphic loci were integrated into the interspecific BC_1_ genetic linkage map [29] using JoinMap V3.0 [31]. The logarithm of odds (LOD) threshold was 5.0. Map distances were measured in centi-Morgans (cM), which were calculated using the Kosambi mapping function [32]. The linkage map was generated using MapChart V2.2 software [33]. QTL mapping of TFs related to fiber quality was performed using the genetic linkage map integrated with the TF markers. The phenotype data of fiber quality and QTL mapping methods were as same as Li et al. [30].

### *In-silico* mapping TFs in two diploid genomes

To verify our genetic mapping results, we blasted the mapped TF sequences against the diploid A_2_ genome of *G*. *arboretum* and D_5_ genome of *G*. *raimondii* (http://www.phytozome.net/cotton.php) with an E-value cut-off 1e-10. Then, the best match for each TF sequence was retained; one TF was only mapped to A_2_ or D_5_ genome rather than both.

### RT-PCR and qRT-PCR analysis

To evaluate differences in TF expression between *G*. *hirsutum* and *G*. *barbadense*, RNAs were extracted from developing fibers at 0 days post-anthesis (DPA), 5 DPA, 10 DPA, 15 DPA, 20 DPA, and 25 DPA, RNAs (4 μg) were reverse-transcribed into cDNA using M-MLV-RT Reverse Transcriptase (Invitrogen). For RT-PCR analysis, PCR was performed in 15 μL of solution containing 25 ng cDNA template, 1.7 x buffer, 3.2 mmol L^-1^ MgCl_2_, 0.42 mmol L^-1^ dNTPs, 0.27 μmol L^-1^ forward primer, 0.27 μmol L^-1^ reverse primer, 1.3 units Taq DNA polymerase, and ddH_2_O to a final volume of 15 μl. The PCR program was as follows: denaturation at 95°C for 5 min; 35 cycles of 94°C (50 s), 58°C (45 s), and 72°C (60 s); and a final extension step of 5 min at 72°C. The qRT-PCR analyses were performed according to the methods described by Munis et al. [34] with minor modifications. Ubiquitin (GenBank acc No.: DQ116441; forward primer, 5’GAAGGCATTCCACCTGACCAAC3’; reverse primer, 5’CTTGACCTTCTTCTTCTTGTGCTTG 3’) served as an internal standard to demonstrate equal amounts of first-strand cDNA in each sample.

## Results

### Primer design and polymorphisms

Primers were designed for the 1,116 TF sequences, and then repeated primers were eliminated by a BLAST analysis. In total, 977 primer pairs were obtained (S1 Table). In the present study, TF primers were screened based on SSCPs. Thirty-four polymorphic primers were obtained, and 37 polymorphic loci were produced with a primer polymorphism rate of 3.48%. These 37 polymorphic loci were from 16 TF families. Seven loci were from the ERF family, and four loci were from the bHLH families. The remaining TF families contained 1–2 polymorphic loci (S1 Table).

### Distribution of TFs in the cotton genome and fiber-related QTLs

**Fig 2:**
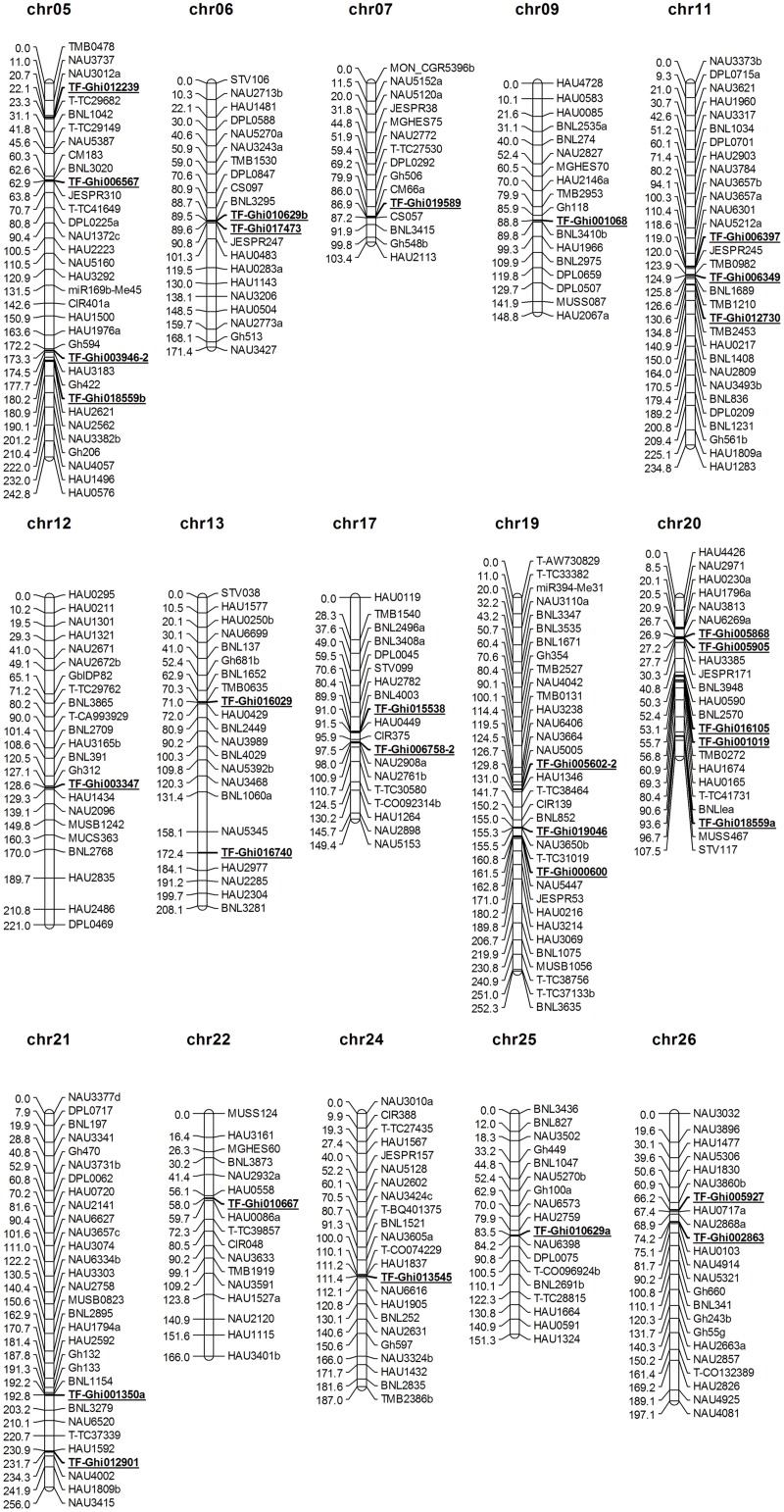
Locations of polymorphic TF loci on the BC_1_ genetic linkage map. TF markers are underlined and bolded. The loci on each chromosome with an average of 10 cM of the original map were selected shown. The top and bottom markers and markers that were closely linked to the TF markers were retained for simplicity.

After linkage analysis, 31 of the 37 TF polymorphic loci were mapped to 15 cotton chromosomes (Chr05, Chr06, Chr07, Chr09, Chr11, Chr12, Chr13, Chr17, Chr19, Chr20, Chr21, Chr22, Chr24, Chr25, and Chr26). Among these chromosomes, 14 TF loci were mapped to 7 chromosomes of the A_T_ genome, and 17 loci were mapped to 8 chromosomes of the D_T_ genome (Fig 2). The 31 TF markers were not equally distributed on the 15 chromosomes. Comparatively, more loci (a total of 15 loci) were mapped to Chr05, Chr11, Chr19, and Chr20. These mapped TF markers belonged to 16 TF families, among which 7 markers were from the ERF family, 4 were from the bHLH family, and 3 were from the WRKY family (S1 Table). As shown in Fig 2, TF-Ghi005868 and TF-Ghi005905 of the ERF family were located on the same chromosome (Chr20) and separated by 0.3 cM. S1 Table indicates that TF-Ghi012239 of the C2H2 family (located on Chr05) and TF-Ghi000600 of the C2H2 family (located on Chr19) were present on homologous chromosomes. In addition, TF-Ghi010629 of the WRKY family has two loci, TF-Ghi010629a and TF-Ghi010629b, which were located on the homologous chromosomes Chr25 and Chr06, respectively. TF-Ghi006349 of the bHLH family and TF-Ghi001350 of the bHLH family were located on the homologous chromosomes, Chr11 and Chr21, respectively.

QTL mapping TF markers associated with fiber quality revealed that only one TF markers, TF-Ghi005602-2 mapped on Chr19, was tightly linked with fiber length with LOD value of 8.70 and explained 12.23% of the phenotypic variance with an additive effect of -0.35. This result may imply that TFs in cotton are involved in developments of many traits rather than fiber development.

### Comparison of the genetically mapped TFs with *in-silico* mapping in two sequenced diploid genomes

**Fig 3:**
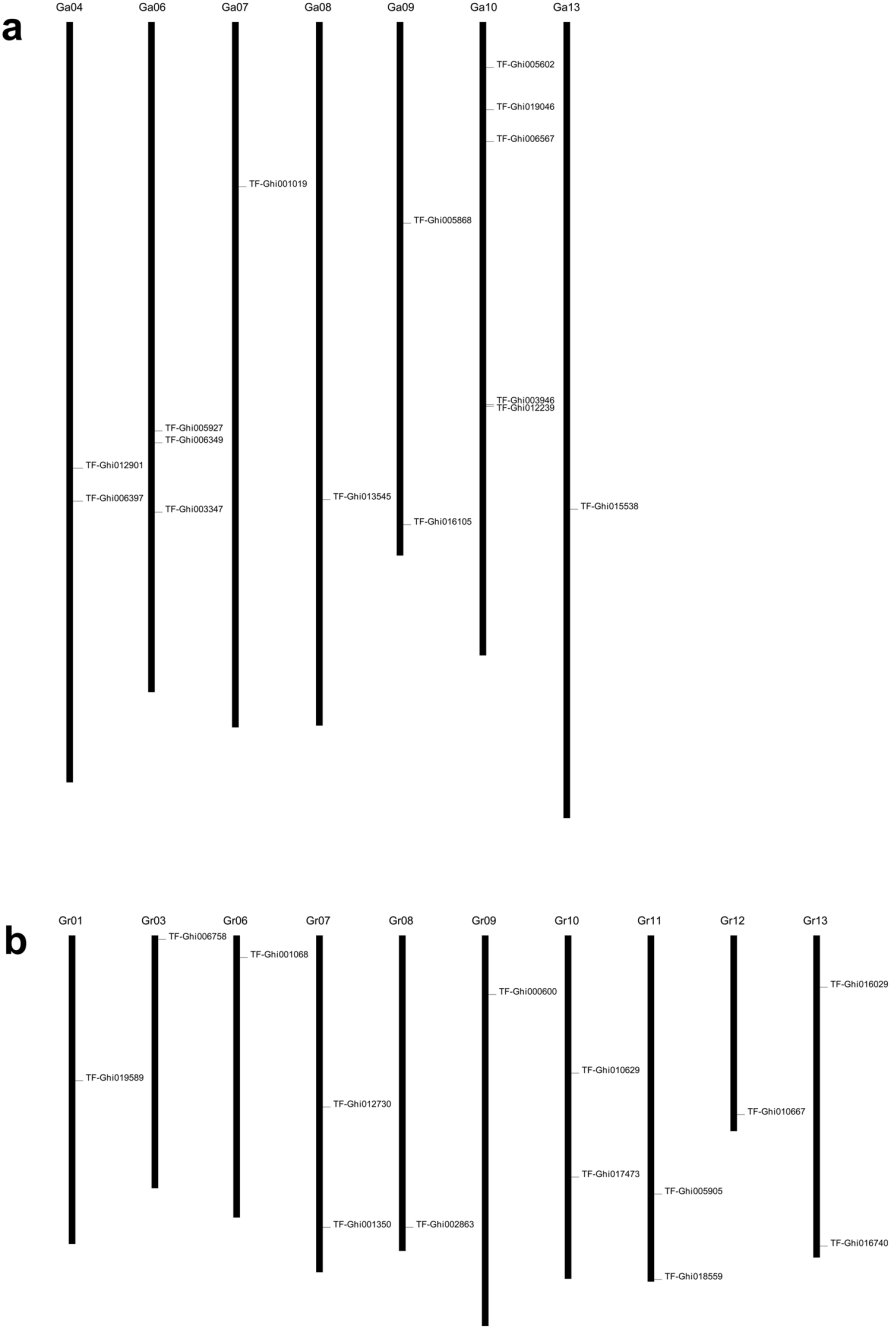
Chromosomal location of TFs in the A_2_ genome of *G*. *arboretum* (a) and D_5_ genome of *G*. *raimondii* (b).

With the availability of cotton genome sequences, we can check the consistency of mapped results with their chromosomal positions by in-silico mapping their sequences to the genome sequence. By unique matching genetically mapped TF sequences to A_2_ and D_5_ genomes, 15 TFs were mapped to 7 chromosomes of A_2_ genome and 14 to 10 chromosomes of D_5_ genome (Fig 3). The in-silico mapping results was different to genetic mapping results. TF-Ghi018559b, mapped on Chr05, was not mapped on the corresponding Ga10, but on Gr11; Gr10 is the corresponding chromosome of Chr20 which is not the homologous chromosome of Chr19. The two loci on Chr06 were not mapped on the corresponding Ga12 but on Gr10; Gr10 is the corresponding chromosome of Chr25 which is the homologous chromosome of Chr06. TF-Ghi019589 on Chr07 was mapped on Gr01, the corresponding chromosome of Chr16 which is the homologous chromosome of Chr07. TF-Ghi001068 on Chr09 was mapped on Gr06, the corresponding chromosome of Chr23 which is the homologous chromosome of Chr09. TF-Ghi006349 on Chr11 was not mapped on the corresponding Ga04, but on Ga06; TF-Ghi012730 was mapped on Gr07, the corresponding chromosome Chr21 which is the homologous chromosome of Chr11. The two loci on Chr13 were mapped on Gr13, the corresponding chromosome Chr18 which is the homologous chromosome of Chr13.

TF-Ghi015538 on Chr17 was mapped on Ga13, the corresponding chromosome Chr13 which is not the homologous chromosome of Chr17. TF-Ghi005602-2 and TF-Ghi019046 on Chr19 was mapped on Ga10, the corresponding chromosome Chr05 which is the homologous chromosome of Chr19. TF-Ghi005868 and TF-Ghi016105 on Chr20 was mapped on Ga09, the corresponding chromosome Chr10 which is the homologous chromosome of Chr20; TF-Ghi001019 was mapped on Ga07, the corresponding chromosome Chr01 which is not the homologous chromosome of Chr20. TF-Ghi013545 on Chr24 was mapped on Ga08, the corresponding chromosome Chr06 which is not the homologous chromosome of Chr24. TF-Ghi005927 on Chr26 was mapped on Ga06, the corresponding chromosome Chr12 which is the homologous chromosome of Chr26.

### RT-PCR and qRT-PCR analysis between mapping parents

**Fig 4:**
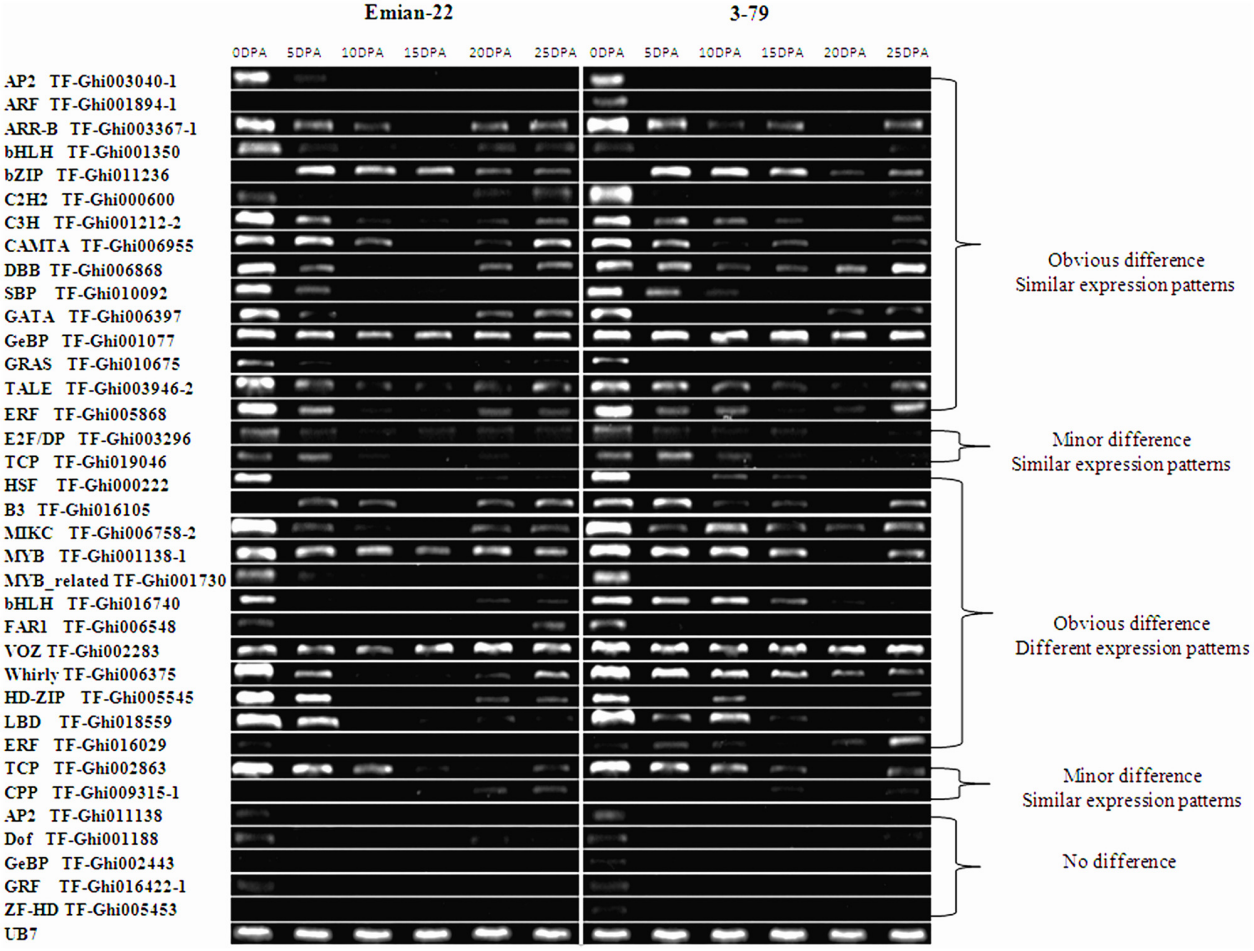
RT-PCR analysis of TFs between mapping parents. The numbers on the top represent fibers at 0 DPA, 5 DPA, 10 DPA, 15 DPA, 20 DPA, and 25 DPA for Emian22 and 3–79. Similar expression trends between Emian22 and 3–79 were classified as similar expression patterns. Apparent differences in expression between Emian22 and 3–79 were classified as different expression patterns. Obvious differences in expression levels between Emian22 and 3–79 were classified as obvious differences. Minor or no differences in expression levels between Emian22 and 3–79 were classified as no difference. Gene primers and their family names are indicated on the left.

One or two markers were randomly selected from each family, and a total of 85 primer pairs from 45 TF families were used for the RT-PCR analysis. Thirty-six TF primer pairs (42.4%) from 31 families were expressed during the cotton fiber stages (Fig 4, Table 2). Among them, 27 displayed clear differences in expression, 4 exhibited minor differences, and 5 showed no differences. Almost all expressed TFs clearly differed between Emian22 and 3–79 at various stages of fiber development (0, 5, 10, 15, 20, and 25 DPA). Five TFs were weakly expressed or not expressed during any stages in either Emian22 or 3–79, and these TFs were defined as having no differences in expression in this study. Furthermore, 17 TFs displayed similar expression patterns, and 14 had different expression patterns. To further confirm the RT-PCR results, four randomly chosen genes belonging to different categories were analyzed (S1 Fig). Consistent results were observed in both the RT-PCR and the qRT-PCR analyses.

**Table 2:** Chromosome localization and RT-PCR analysis of TFs.

c	Primer name	Chromosome	Genome	Results of RT-PCR
AP2	TF-Ghi003040-1	-	-	1
	TF-Ghi011138	-	-	0
ARF	TF-Ghi001894-1	-	-	1
	TF-Ghi009171-1	-	-	-
ARR-B	TF-Ghi001951	-	-	-
	TF-Ghi003367-1	-	-	1
B3	TF-Ghi016105	Chr 20	D_T_	1
	TF-Ghi004897	-	-	-
BBR/BPC	TF-Ghi013423	-	-	-
bHLH	TF-Ghi001350	Chr 21	D_T_	1
	TF-Ghi016740	Chr 13	A_T_	1
bZIP	TF-Ghi011236	-	-	1
	TF-Ghi000928	-	-	-
C2H2	TF-Ghi000600	Chr 19	D_T_	1
	TF-Ghi012239	Chr 05	A_T_	-
C3H	TF-Ghi001212-2	-	-	1
	TF-Ghi002804-1	-	-	-
CAMTA	TF-Ghi006955	-	-	1
	TF-Ghi015073	-	-	-
CO-like	TF-Ghi011118	-	-	-
	TF-Ghi012473-2	-	-	-
CPP	TF-Ghi005234-2	-	-	-
	TF-Ghi009315-1	-	-	0
DBB	TF-Ghi006868	-	-	1
	TF-Ghi007689	-	-	-
Dof	TF-Ghi001188	-	-	0
	TF-Ghi006567	Chr 05	A_T_	-
E2F/DP	TF-Ghi003296	-	-	0
	TF-Ghi010249	-	-	-
EIL	TF-Ghi005277	-	-	-
	TF-Ghi012794	-	-	-
ERF	TF-Ghi005868	Chr 20	D_T_	1
	TF-Ghi016029	Chr 13	A_T_	1
FAR1	TF-Ghi001781	-	-	-
	TF-Ghi006548	-	-	1
G2-like	TF-Ghi003043	-	-	-
	TF-Ghi010667	Chr 22	D_T_	-
GATA	TF-Ghi001019	Chr 20	D_T_	-
	TF-Ghi006397	Chr 11	A_T_	1
GeBP	TF-Ghi001077	-	-	1
	TF-Ghi002443	-	-	0
GRAS	TF-Ghi002005	-	-	-
	TF-Ghi010675	-	-	1
GRF	TF-Ghi005602-2	Chr 19	D_T_	-
	TF-Ghi016422-1	-	-	0
HB-other	TF-Ghi011486	-	-	-
	TF-Ghi015378	-	-	-
HD-ZIP	TF-Ghi005545	-	-	1
	TF-Ghi012730	Chr 11	A_T_	-
HSF	TF-Ghi000222	-	-	1
	TF-Ghi011976	-	-	-
LBD	TF-Ghi007885	-	-	-
	TF-Ghi018559	Chr 05, Chr 20	A_T_, D_T_	1
MIKC	TF-Ghi006758-2	Chr 17	D_T_	1
	TF-Ghi015198-1	-	-	-
MYB	TF-Ghi001138-1	-	-	1
	TF-Ghi005417	-	-	-
MYB_related	TF-Ghi001730	-	-	1
	TF-Ghi007525	-	-	-
NAC	TF-Ghi009255	-	-	-
	TF-Ghi009258	-	-	-
NF-YA	TF-Ghi004749	-	-	-
	TF-Ghi004977	-	-	-
NF-YB	TF-Ghi008239	-	-	-
	TF-Ghi019211	-	-	-
NF-YC	TF-Ghi012195	-	-	-
	TF-Ghi014911	-	-	-
Nin-like	TF-Ghi001151	-	-	-
	TF-Ghi015538	Chr 17	D_T_	-
RAV	TF-Ghi013999	-	-	-
S1Fa-like	TF-Ghi013563	-	-	-
SBP	TF-Ghi010092	-	-	1
SRS	TF-Ghi002427	-	-	-
TALE	TF-Ghi003946-2	Chr 05	A_T_	1
	TF-Ghi012410	-	-	-
TCP	TF-Ghi002863	Chr 26	D_T_	0
	TF-Ghi019046	Chr 19	D_T_	0
Trihelix	TF-Ghi010211	-	-	-
	TF-Ghi011360	-	-	-
VOZ	TF-Ghi002283	-	-	1
	TF-Ghi007684	-	-	-
Whirly	TF-Ghi006375	-	-	1
WOX	TF-Ghi006162	-	-	-
	TF-Ghi011650	-	-	-
WRKY	TF-Ghi003347	Chr 12	A_T_	-
	TF-Ghi003889	-	-	-
YABBY	TF-Ghi001411	-	-	-
	TF-Ghi017422	-	-	-
ZF-HD	TF-Ghi001068	Chr 09	A_T_	-
	TF-Ghi005453	-	-	0

-: TF was not mapped on the chromosomes, and expression was not detected.

0: No differences in expression.

1: Obvious difference in expression.

## Discussion

### Low level of polymorphisms of the TF markers

TFs, as an important type of trans-acting factor, are extensively involved in the development of plants and animals. Various TFs are related to cotton fiber development [3,16,35,36]. A large number of TF functions have been verified. However, few genetic mapping studies of TFs have been performed in cotton [37]. Therefore, we downloaded TF sequences reported in the Plant Transcription Factor Database V2.0 (http://planttfdb.cbi.edu.cn/) (Table 1) and designed specific primers based on the motifs of the TF genes. Thus, the primers developed in the present study were TF-specific. In contrast, the simple sequence repeats (SSRs) primers derived from the TF sequences [37] may not have been TF-specific.

To detect additional polymorphisms in the TF markers, SSCP was applied. We discovered a very low rate of primer polymorphisms in the TF markers (3.48%), which was potentially attributed to the highly conserved nature of TFs. The results also indicated that the genes that were compared between *G*. *hirsutum* and *G*. *barbadense* are highly conserved. Although the primer polymorphism rate was low, the TF markers examined in this study exhibited more polymorphisms than other TF markers. Li et al. [37] used SSRs designed from 1,116 *G*. *hirsutum* TFs in an analysis of polymorphisms and revealed polymorphism ratios of 1.6%, 2.1%, and 2.3% in the (Yumian 1×CCRI35) F_2:6_, (Yumian 1×T586) F_2:7_, and (Yumian 1×7235) F_2:6_ populations, respectively. The higher rate of polymorphisms detected in the present study may have been caused by SSCP, which can detect minor difference between sequences [38]. This result may also be caused by differences between the interspecific and intraspecific populations.

### Genetic mapping TFs in cotton

To date, some genetic mapping analyses of TFs have been reported. SNP primers of the MYB family were mapped on the cotton chromosome by An et al. [39]. Myb1Gbmt_238 is located on Chr13, Myb1Gb_500 is located on Chr18, and Myb2Gb_204 is located on Chr8. Guo et al. [40] also mapped TFs of the MYB family on cotton chromosomes; for example, MYB38 is located on Chr16. However, in the present study, TF markers of the MYB family were not mapped on the interspecific linkage map. In 2012, SSR primers designed from the same 1,116 *G*. *hirsutum* TFs were mapped on cotton chromosomes by Li et al. [37]. Unfortunately, due to different marker develop strategy, no common TFs were mapped.

In this study, 31 polymorphic TF loci were mapped to 15 chromosomes. Among them, 14 TF loci were mapped to 7 chromosomes of the A_T_ genome and 17 loci on 8 chromosomes of the D_T_ genome (Fig 2). These loci were clearly evenly distributed between the A_T_ and the D_T_ genome. However, Chr05, Chr11, Chr19, and Chr20 contained 15 loci that accounted for 48.4% of the total mapped TF loci. Therefore, genetic mapping revealed preferential distribution of TF loci on cotton chromosomes (Fig 2). Comparison of chromosomal location of TF loci revealed that TFs from the same family mapped to homologous cotton chromosomes.

Comparison of the genetically mapped TFs with in-silico mapping results in two sequenced diploid genomes showed that some TFs were not mapped on their corresponding chromosomes in the diploid genome, but on their homologous chromosomes’ corresponding chromosomes in the other diploid genome. It is reasonable that many genes are duplicated on the homologous chromosomes in the tetraploid genome. However, some TFs were not mapped on their corresponding chromosomes or their homologous chromosomes’ corresponding chromosomes in the diploid genomes; the reason may be that these genes translocate after polyploidization.

### Differences in the expression of TFs between *G*. *hirsutum* and *G*. *barbadense* during fiber development

Cotton is an important cash crop. Therefore, improvements in yield, fiber quality, and disease resistance are areas of focus in cotton genetics and breeding. A number of studies have indicated that various TFs are involved in fiber development. Therefore, in the present study, we used RT-PCR analysis to compare TFs between *G*. *hirsutum* and *G*. *barbadense* during fiber development.

We discovered dynamic expression of TFs during various stages of fiber development in *G*. *hirsutum* and *G*. *barbadense*. The most expressed TFs (75%) from 25 families exhibited significantly different expression levels during different stages between parents. Further studies of TFs showing different expression patterns between *G*. *hirsutum* and *G*. *barbadense* may be very helpful for understanding differences in fiber quality between the two species. Unique expression patterns may be associated with a particular function. Additional studies are required to determine the functions and mechanisms of action of these TFs.

## Supporting Information

S1 Fig qRT-PCR analysis of TFs between mapping parents. The expression levels of Emian22 and 3–79 are presented. “*” represents *P*≤0.05, and “**” represents *P*≤0.01. The following primers were used: A, TF-Ghi001138-1; B, TF-Ghi003946-2; C, TF-Ghi005868; D, TF-Ghi016740.
(TIF)

S1 Table Characteristics of the 977 TF primers used in this study. √: Polymorphic primers. ×: Non-polymorphic primers. -: The primer was not mapped on the chromosome.
(XLS)
