# Supplementary figures and images for: Genetic Mapping and Comparative Expression Analysis of Transcription Factors in Cotton
Source: PLoS One. 2015 May 6;10(5):e0126150. doi: 10.1371/journal.pone.0126150 (PMC4422734; doi:10.1371/journal.pone.0126150)

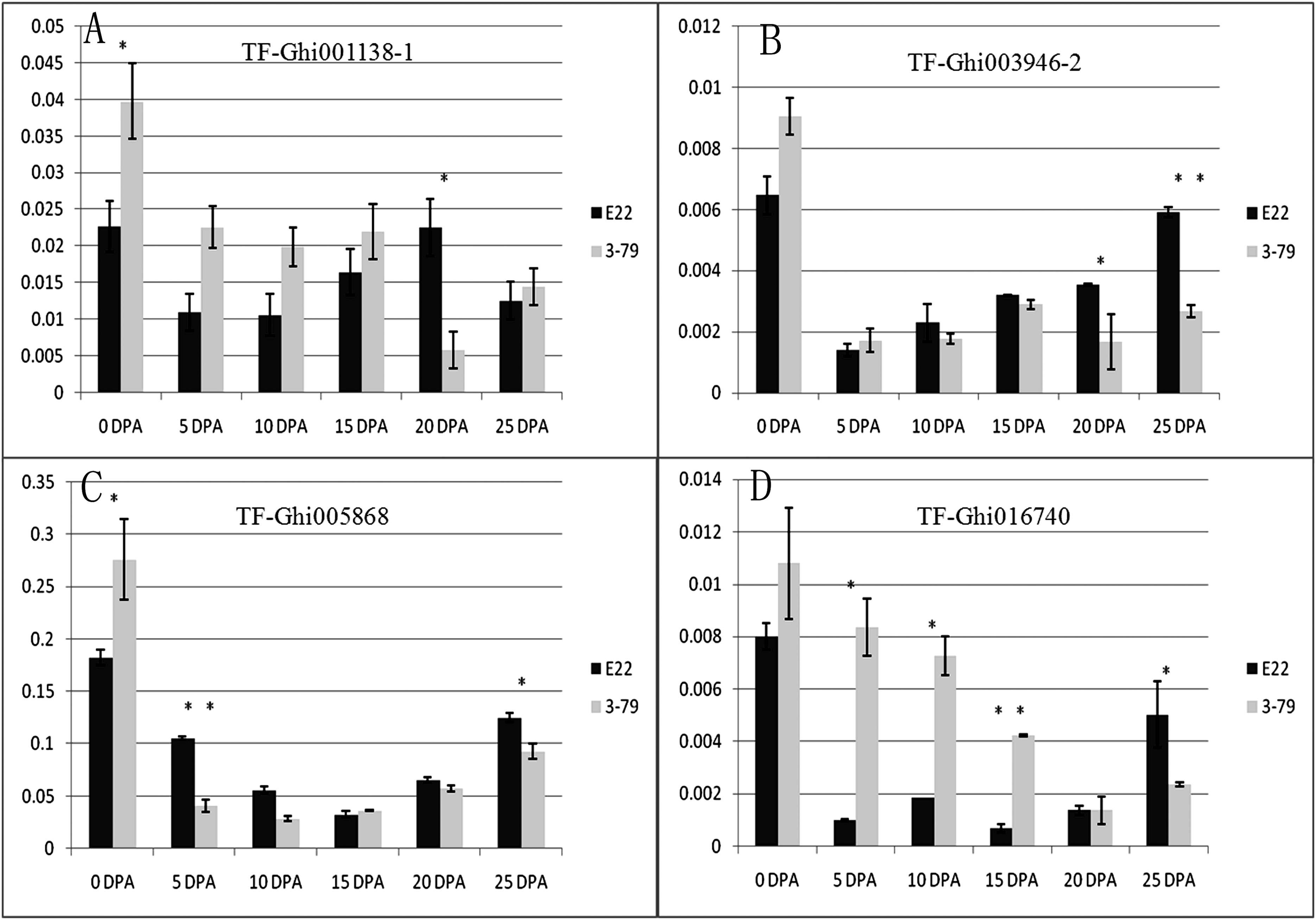

Supplement: S1 Fig — The expression levels of Emian22 and 3–79 are presented. “*” represents P≤0.05, and “**” represents P≤0.01. The following primers were used: A, TF-Ghi001138-1; B, TF-Ghi003946-2; C, TF-Ghi005868; D, TF-Ghi016740. (TIF) [file pone.0126150.s001.tif]
